# Supplementary material for: Asymmetric Synthesis of Quaternary Hydantoins via a Palladium-Catalyzed Aza-Heck Cyclization
Source: J Am Chem Soc. 2025 Nov 14;147(49):44692–8. doi: 10.1021/jacs.5c16022 (PMC12703750; doi:10.1021/jacs.5c16022)
Supplement: Supplementary file 2 [file ja5c16022_si_002.zip › All NMR FID Files/3/3_ProtonNMR/1/pdata/1/email_EM02-247_1_1.pdf]

PROTON8 CDCl3 /opt/nmrdata emeck 11

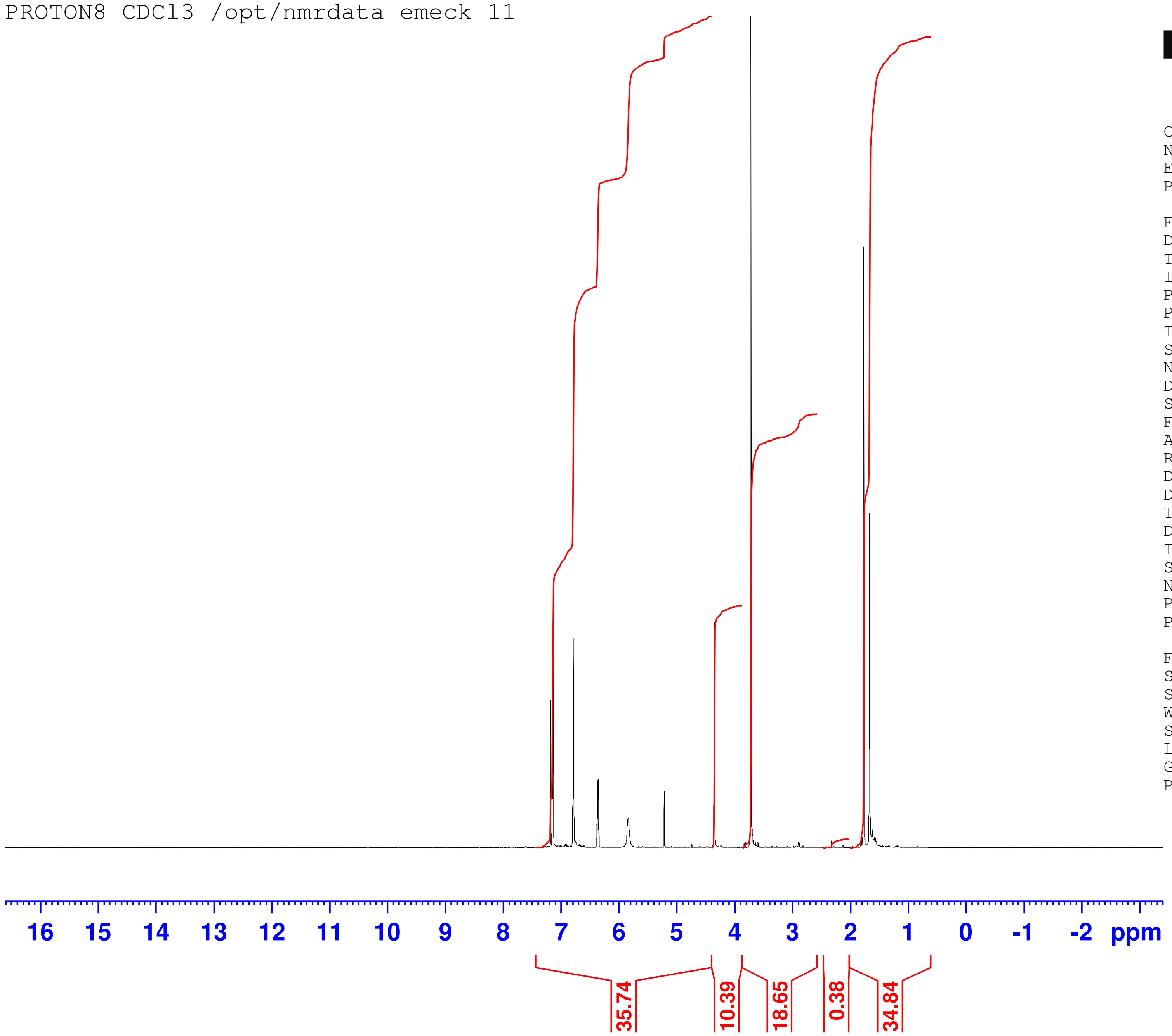

Current Data Parameters

NAME	EM02-247
EXPNO	1
PROCNO	1

F2 - Acquisition Parameters

Date_	20220609
Time	12.07 h
INSTRUM	spect
PROBHD	Z114607_0069 (
PULPROG	zg30
TD	65536
SOLVENT	CDCl3
NS	16
DS	2
SWH	12019.230 Hz
FIDRES	0.366798 Hz
AQ	2.7262976 sec
RG	57
DW	41.600 usec
DE	11.53 usec
TE	300.0 K
D1	1.00000000 sec
TD0	1
SFO1	600.3240221 MHz
NUC1	1H
P1	11.15 usec
PLW1	26.00000000 W

F2 - Processing parameters

SI	65536
SF	600.3200553 MHz
WDW	EM
SSB	0
LB	0.30 Hz
GB	0
PC	1.00
